# Microbiome distinctions between the CRC carcinogenic pathways

**DOI:** 10.1080/19490976.2020.1854641

**Published:** 2021-01-15

**Authors:** Lauren DeDecker, Bretton Coppedge, Julio Avelar-Barragan, William Karnes, Katrine Whiteson

**Affiliations:** a School of Medicine, University of California, Irvine, California, USA; b School of Biological Sciences, University of California, Irvine, California, USA

**Keywords:** Colorectal cancer, microbiome, serrated, adenoma, carcinogenesis

## Abstract

Colorectal cancer (CRC) is the third most commonly diagnosed cancer, the third leading cause of cancer-related deaths, and has been on the rise among young adults in the United States. Research has established that the colonic microbiome is different in patients with CRC compared to healthy controls, but few studies have investigated if and how the microbiome may relate to CRC progression through the serrated pathway versus the adenoma-carcinoma sequence.

Our view is that progress in CRC microbiome research requires consideration of how the microbiome may contribute to CRC carcinogenesis through the distinct pathways that lead to CRC, which could enable the creation of novel and tailored prevention, screening, and therapeutic interventions. We first highlight the limitations in existing CRC microbiome research and offer corresponding solutions for investigating the microbiome’s role in the adenoma-carcinoma sequence and serrated pathway. We then summarize the findings in the select human studies that included data points related to the two major carcinogenic pathways. These studies investigate the microbiome in CRC carcinogenesis and 1) utilize mucosal samples and 2) compare polyps or tumors by histopathologic type, molecular/genetic type, or location in the colon.

Key findings from these studies include: 1) *Fusobacterium* is associated with right-sided, more advanced, and serrated lesions; 2) the colons of people with CRC have bacteria typically associated with normal oral flora; and 3) colons from people with CRC have more biofilms, and these biofilms are predominantly located in the proximal colon (single study).

## Introduction

Colorectal cancer (CRC) is the third most commonly diagnosed cancer, the third leading cause of cancer-related deaths^1^, and is increasing in young adults in the United States.^2^ It is estimated that over 50% of the screening-age population (≥50 years) have one or more precancerous adenomas or polyps.

The Cancer Genome Atlas project, utilizing extensive genomic and transcriptomic characterization of colorectal cancer, has proposed the classification of CRC into two major groups: tumors with microsatellite instability (MSI) and tumors with chromosomal instability.^3^ Cancers with microsatellite instability largely result from defective DNA mismatch repair caused by inactivating mutations or epigenetic silencing of mismatch repair genes such as the *MLH1* tumor suppressor gene. Epigenetic silencing frequently occurs due to CpG island promoter methylation of *MLH1*. A majority of tumors arising from this pathway, termed the serrated pathway, also have *BRAF V600E* mutations, and a minority have DNA polymerase Epsilon or Delta 1 mutations. In contrast, cancers with chromosomal instability have alterations in chromosome number and activating mutations in oncogenes *K-ras, PIK3CA* or inactivating mutations in tumor suppressor genes *Apc, p53* and *SMAD4*. These are the hallmark alterations seen in tumors that arise by the adenoma-carcinoma sequence, first characterized by Fearon and Vogelstein.^4^

While the majority of CRC develops through the adenoma-carcinoma sequence, up to a third of CRC develops via the serrated pathway.^5^ Precursor lesions of the serrated pathway or an alternate pathway include a broad group of serrated polyps, including benign hyperplastic polyps (HPs), precancerous traditional serrated adenomas (TSAs) and sessile serrated polyps (SSPs). TSAs may exhibit elements of the adenoma-carcinoma sequence such as *K-ras* mutation, or may have *BRAF* mutation^6^ and CpG island hypermethylation (a signature of the sessile serrated pathway^7^). TSAs are typically found in the left (distal) colon.^8^ In contrast, SSPs rarely demonstrate elements of the chromosomal instability pathway but frequently have *BRAF* mutations and appear to progress toward dysplasia and carcinoma as a result of microsatellite instability due to *MLH1* promoter CpG island hypermethylation. SSPs are found in the right (proximal) side of the colon 80% of the time,^9^ consistent with findings that tumors characterized by *BRAF* mutations, microsatellite instability and CpG island hypermethylation phenotype (CIMP) have shown a linear increase in frequency from distal to proximal colon.^10^ A summary of the major pathways of carcinogenesis can be seen in Figure 2.

It is not yet known what factors influence the progression of a CRC precursor through the adenoma-carcinoma sequence, serrated pathway or an alternate pathway. It is tempting to hypothesize that the microbiome may play a role, but most research has focused on the microbiome alterations in patients with CRC or precursor polyps without further specification of the histopathologic, genetic, or epigenetic type. This review will briefly summarize the proposed mechanisms of how the microbiome contributes to CRC from existing research, the most common limitations in this research, and offer corresponding solutions. We then summarize the key findings in the select studies that have added data points related to the major carcinogenic pathways to CRC.

## Mechanisms of the microbiome in CRC carcinogenesis

**Figure 1.**
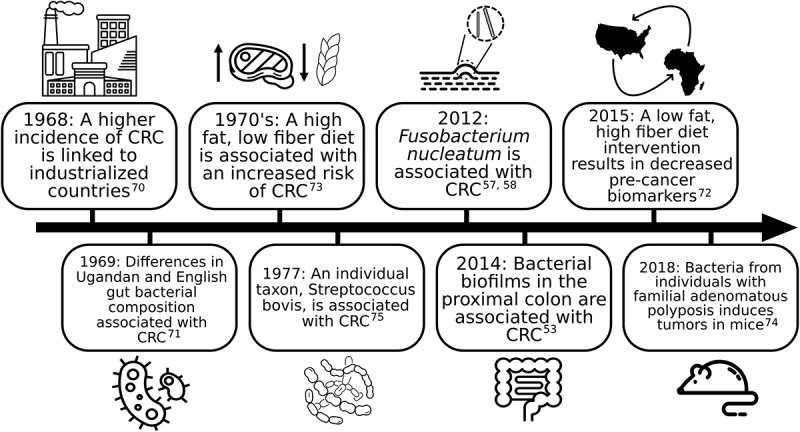
Landmark research on the microbiome and colorectal cancer

Research has established the importance of diet and lifestyle in CRC carcinogenesis.^11^ The microbiome, intimately related to the environmental factors linked to CRC, has been postulated to play a role in CRC carcinogenesis since the 1960s. While single bacterial strains have been associated with CRC Table 1, the current belief is that intestinal microbial dysbiosis and a subsequent inappropriate or altered immune response can confer a predisposition to chronic inflammation,^17–19^ which is known to contribute to the development of disease and cancer. Microbes may contribute to genetic and epigenetic alterations via the production of superoxide radicals and genotoxins and toll-like receptor mediated induction of carcinogenic pathways.^15,20–22^ Diets rich in fiber have been associated with a decreased risk of CRC,^23^ which may be due to the production of butyrate by colonic bacteria.^24,25^ Studies on butyrate have shown that it reduces inflammation and can inhibit growth and induce apoptosis in cancer cells.^26^ An imbalance of butyrate, folate, and biotin-producing bacteria^27^ could contribute to carcinogenesis as these molecules are involved in epithelial proliferation either directly or epigenetically.^28–30^ Further, secondary bile acids may be carcinogenic by acting as mutagens, eliciting reactive oxygen species, and increasing NF-kappa B activation, resulting in inflammation.^31^ Additionally, a diet low in fiber leaves the colon devoid of Microbiota Accessible Carbohydrates^32^ and open for bacteria to feed on the protein-rich mucus layer that protects the colon epithelium.^33^ It is possible that the microbial switch from metabolizing carbohydrates to proteins generates inflammatory side products and loss of the protective mucus layer results in direct contact of bacteria with the epithelium. This direct contact has been proposed as a step in inciting cellular changes or inflammation in the colon epithelium.^34^ Landmark studies on the microbiome and CRC are summarized in Figure 1.

**Figure 2.**
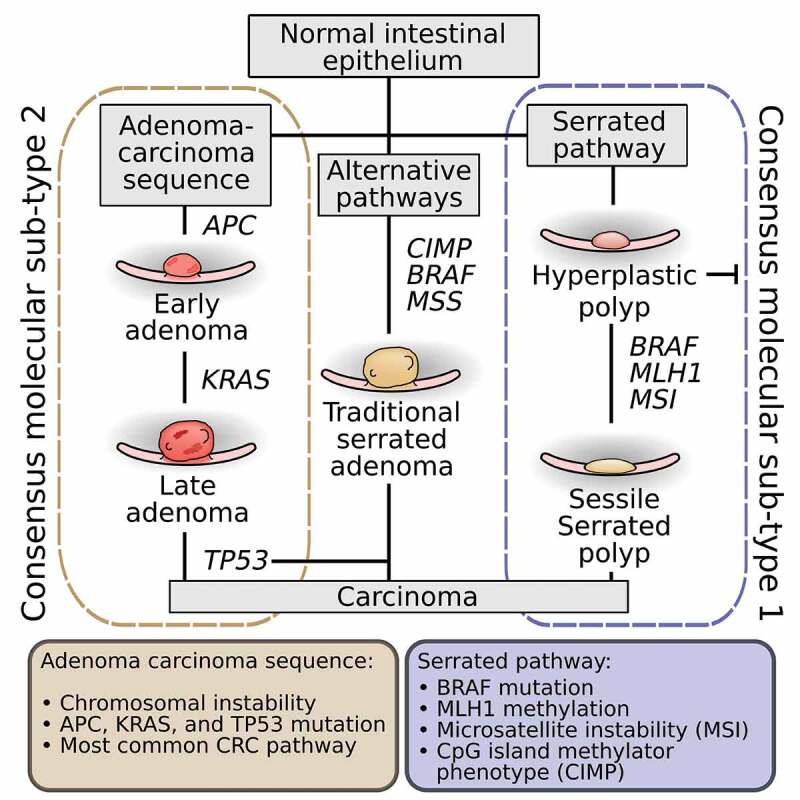
The pathways of CRC carcinogenesis.(4,6,7).

**Table 1.** The proposed mechanisms of individual bacteria implicated in colorectal cancer

Bacteria	Proposed CRC Contribution Mechanism
*Enterococcus faecalis*	Produces reactive oxygen species that can cause DNA damage, contributing to chromosomal instability.^12^
*Enterotoxigenic Bacteroides fragilis* (ETBF)	Produces the *B. fragilis* toxin, which is directly genotoxic and cleaves the tumor suppressor protein E-cadherin, resulting in enhanced Wnt/beta-catenin and NF-kB signaling leading to increased mucosal permeability and colonocyte proliferation. ETBF may also increase tumorigenesis through its association with upregulated Interleukin-17, which activates STAT3, and T-helper cell 17 inflammatory response.^13^
*Streptococcus gallolyticus*	*S. gallolyticus* in the blood has long been a red flag for carcinoma of the colon. It thrives in environments seen in colonic tumors, is able to translocate through the epithelium, and is associated with enhanced inflammatory signaling.^14^
*Escherichia coli*	Particular strains can secrete colibactin toxin and induce double stranded DNA breaks in mice,^15^ and may also downregulate DNA mismatch repair proteins.
*Fusobacterium nucleatum*	Generates the FadA adhesin protein which allows it to bind cell E-cadherin and activate Wnt signaling.^16^ It also appears to recruit immune cells to the tumor environment and upregulate inflammatory genes.

## Research limitations and solutions

To date, most studies have focused on comparing the intestinal microbiome between healthy individuals and those with CRC tumors or adenomas using stool samples. Research has demonstrated that there are differences in the microbiota of colons with CRC, such as an increase in *Prevotella^35^* and *Fusobacteria*,^36–39^ reduction in butyrate-producing bacteria,^40–42^ *Bifidobacteria*,^43^ and overall diversity.^41^ However, some of these findings are inconsistent across the literature, potentially due to differing study design and a number of common limitations found in CRC microbiome studies. Additionally, these microbial differences may therefore be consequential to the altered biochemical environment of CRC tumor mucosa^44^ and unrelated to processes that triggered the development of CRC.

We suggest five limitations to existing human studies and present potential corresponding solutions. The first frequent limitation among human studies is the sole use of stool samples. This may be problematic as the composition of mucosa-associated microbes which adhere to the intestinal epithelia has been shown to be different from microbes found in the lumen, which are more likely found in stool.^40–43^ Stool samples have a blend of the microbial communities present in the colon and are unable to provide the same location information as localized samples from the mucosa. Given that microbes in close contact with the epithelium may have greater potential to influence progression into CRC, studying mucosa-associated microbes by obtaining mucosal samples from precursor lesions and tumors may provide more useful data.

Second, the majority of studies have compared the microbiome of healthy individuals and patients with CRC tumors. The list of microbes that has been found to be enriched or deficient in CRC tumors may be related to the altered environment of the tumor rather than the microbe’s involvement in CRC carcinogenesis. CRC tumors have been found to have decreased glucose, lower pH, and elevated amino acids and fatty acids.^44^ To determine if and how the microbiome may contribute to CRC carcinogenesis, we suggest that sample collection be broadened to include precursor lesions in the sequential stages of carcinogenesis as well as CRC tumors. Examination of precursor lesions is important for elucidating early changes in the microbiome that may incite or accelerate the progression of CRC carcinogenesis.

A third limitation is that many of the studies that examine precursor lesions do not differentiate based on polyp type (adenoma vs. SSP vs. TSA). Given that CRC carcinogenesis proceeds predominantly through two unique pathways, it is possible that the microbiome may contribute to the incitement and/or progression of each pathway in specific ways. We believe that changes in microbial populations specific to polyp and tumor type should be investigated, which can be done using genetic, molecular, or histopathological characterization of colorectal polyps and tumors.

**Figure 3.**
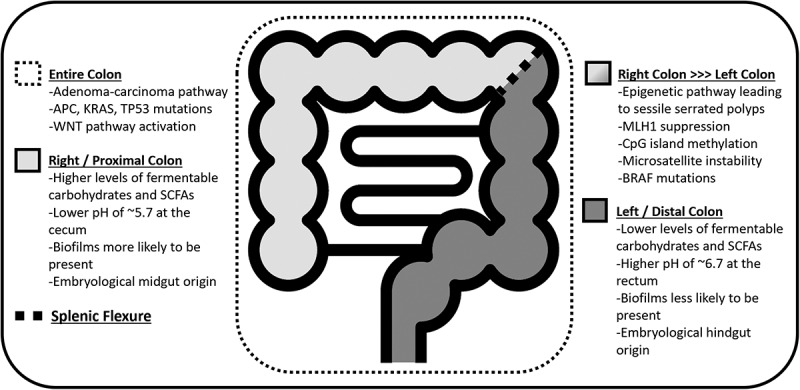
Biogeographical differences and CRC pathways throughout the colon.(53,76,80)

A fourth limitation is that when mucosa has been sampled, the information regarding the site in the colon where the sample was collected, a vital piece of metadata, is often not reported. Location data are important because while the adenoma-carcinoma pathway is found throughout the colon, the epigenetic sessile serrated pathway is more uniquely right sided Figure 3. For example, several groups have demonstrated that *Fusobacteria* is associated with right-sided colonic lesions with features of the serrated pathway.^36–38^ We suggest that additional data points of location in the colon be added during the sample collecting process, such as if the sample was obtained from the proximal or distal colon, or rectum. The differences in terms of anatomical location within the colon and characteristics of each pathway are summarized in Figures 2 and 3.

A fifth limitation is that some studies have been limited in the type of sequencing used to characterize the microbiome. The prevailing approach is 16S rRNA amplicon sequencing, which focuses on an individual, universal marker gene. 16S rRNA gene sequencing can elucidate which bacteria are present in a sample but does not include information about specific metabolic capacities of individual strains or species. The increased resolution provided by whole community shotgun metagenomic sequencing may in part explain why a recent meta-analysis of 16S rRNA fecal microbiomes from CRC patients failed to find biomarkers of CRC,^45^ while subsequent shotgun sequencing meta-analyses detected increased protein and mucin catabolism genes and reduced carbohydrate degradation genes.^46^ Thus, we recommend that future studies consider using shotgun sequencing for their sample analysis. In addition, it is critical that microbiome researchers use consistent standards, such as having positive and negative controls as part of every study and using spike-in standards to get semi-quantitative data.^47^ It is also beneficial to use consistent taxonomic resolution. Research data and analyses should be open access to support rigorous and reproducible science.

## Microbiome of the serrated pathway vs. the adenoma-carcinoma sequence

It is our belief that human studies that use mucosal samples and differentiate polyp or tumor samples by either location in the colon, histopathologic type, or molecular/genetic type will provide key information on how the microbiome may 1) contribute to CRC carcinogenesis, and 2) play specific roles in the two major pathways of CRC carcinogenesis. We found that the number of studies that investigated the mucosal microbiome and differentiated lesions in any of the aforementioned ways was very limited. Four studies included data on lesions classified by molecular/genetic type,^36,48–50^ seven included location in the colon,^36,38,49–53^ and three by histopathologic type.^36–38^ These 9 studies are summarized in Table 2, with key findings discussed below. There is a notable gap in existing research with regards to differentiating between TSAs and SSPs in addition to adenomas. One of the studies that categorized lesions by genetic mutation revealed some interesting patterns that could be theoretically extrapolated to the different polyp types. Burns et al. found that lesions with *Apc* mutations (seen in the adenoma-carcinoma sequence) have an increase in *Finegoldia*, which is an opportunistic pathogen at sites of epithelial disruption. Lesions with mutations in *KMTC2*, which is commonly mutated with *K-ras* (part of the adenoma-carcinoma sequence and frequently mutated in TSAs) could be predicted by the abundance of *Ruminococcus*, which has been linked to IBD and CRC.^48^ Another study found that SSPs with dysplasia, but not without dysplasia or adenomas, had characteristic *Clostridium perfringens* infection. They hypothesized that *C. perfringens* may enhance carcinogenesis via Yes-associated protein activation.^54^

**Table 2.** Microbiome studies differentiating by CRC carcinogenic pathway or location histopathologic type

Author	Sample type	Location in colon	Histopathologic type	Molecular/Genetic type	Sample source
Flemer et al.^51^ 2017	Mucosa, stool	X			On and off tumor
Gao et al.^52^ 2015	Mucosa	X			On and off tumor
Dejea et al.^53^ 2014	Mucosa	X			Biofilms on and off tumors and precursor lesions
Burns et al.^48^ 2016	Mucosa			X	On and off tumor
Ito et al.^36^ 2015	Mucosa	X	X	X	Tumors and precursor lesions
Purcell et al.^49^ 2017	Mucosa	X		X	Tumors
Hale et al.^50^ 2018	Mucosa	X		X	On and off tumor
Park et al.^37^ 2016	Mucosa		X		Precursor lesions, tumors
Yu et al.^38^ 2016	Mucosa	X	X		*F. nucleatum* in tumors and precursor lesions

## Biofilm presence in left vs. right colon

Studies by Flemer et al., Gao et al., and Dejea et al. compared left versus right CRC microbiota. These studies revealed that there are significantly different bacteria in patients with CRC versus those without, and in proximal versus distal CRC.^51,52^ More importantly, different clusters of bacteria were associated with different gut mucosa gene expression patterns, which could provide the link between colon microenvironments and their unique microbial inhabitants and ensuing pathogenesis. The most significant finding was in the study by Dejea et al.^53^ examining the presence of biofilms on adenomas and carcinomas. Biofilms contain an array of microorganisms that are adherent to one another as well as a surface by way of extracellular matrix produced by bacteria. The polysaccharide layer promotes bacterial survival in nutrient poor conditions and allows the inhabitants to exchange metabolites with the environment external to the biofilm. Biofilms may create pro-carcinogenic environments that are as important to the development of cancer as the specific taxa present. Dejea et al. found that biofilms were predominantly found on proximal CRC and adenomas, and tumors with biofilms displayed bacterial invasion. Biofilm-covered epithelial cells were shown to have decreased E-cadherin (a transmembrane inter-cellular binding protein) leading to increased mucosal permeability, increased Interleukin-6 (an inflammatory mediator) and increased STAT-3 activation leading to proliferation. In theory, biofilm formation and bacterial invasion could allow bacteria to persistently interact with the epithelium and lead to chronic inflammation and carcinogenesis. Of note, the aforementioned changes were also seen in a subset of healthy individuals with biofilms, begging the question of whether biofilm presence can contribute to the incitement of CRC carcinogenesis. A subsequent study by Johnson and Dejea found that biofilms contained increased levels of polyamine metabolites (molecules in all eukaryotic cells essential for growth) associated with cellular proliferation and colon cancer.^55^ Specifically, the level of a polyamine metabolite that has been a proposed marker for early-stage CRC^56^ was elevated in tumors regardless of biofilm presence and was further elevated in the presence of biofilms on tumor and normal tissue. While biofilms are predominantly present on right-sided colon cancers, the increased acetylated polyamine metabolites on biofilm-positive cancer and paired normal tissues on the left side suggests that biofilm presence, not colon location, is what alters polyamine metabolism leading to metabolites associated with CRC. The association of biofilms with proximal CRC raises the question of whether biofilms may specifically contribute to the sessile serrated pathway. Further research is needed to define the genetic and histopathologic type of polyps and tumors that biofilms are found on.

## *Fusobacterium* and serrated pathway lesions

Many studies have implicated the genus *Fusobacterium* in CRC, as early as the 2012 studies by Kostic et al.^57^ and Castellarin et al.^58^ Some studies, such as the reproducibility study by Repass have not found the same association, but these mixed findings could be related to varying population samples, sample types, and sequencing methods.^59^ Our analysis of the included studies revealed a clear association between *Fusobacterium* and lesions that were proximal, of higher histological grade, and with features of the serrated pathway. The study by Yu et al. found that *Fusobacterium* was found more frequently in SSPs than adenomas, and in proximal more than distal CRC. While Ito et al. did not detect a difference in *Fusobacterium* between adenomas, TSAs, or SSPs, his group did find that the rate of *Fusobacterium* positive SSPs increased when moving from distal to proximal colon.^36,39^ Studies by Park and Ito found *Fusobacterium* to be more associated with CRC than less advanced lesions, with *F. nucleatum’s* presence increasing as the histological grade increased, which could be the result of new microenvironments of CRC tumors.^36,37^ SSPs often have a mucus cap and overexpress mucin forming genes such as *MUC6, MUC5aC, MUC17*, and *MUC2*, which has been associated with increased metastasis.^60^ It is possible that this mucus cap can help *Fusobacterium* and other bacteria survive. Mucus caps may function similar to biofilms; however, Yu et al.^38^ found that *Fusobacterium* presence and its ability to invade the mucosa did not depend on biofilm presence. All of the studies differentiating microbial populations by molecular or genetic mutation found *Fusobacterium* to be associated with lesions characterized by features of the serrated pathway including mismatch repair (MMR) deficiency, *MLH1* methylation, CpG island methylator phenotype, or high microsatellite instability.^36,49,50^ Interestingly, Hale et al. found that MMR status was a stronger predictor of the microbe community variance than even location in the colon and off versus on tumor. Of note, these findings are associations, and whether *Fusobacterium* is a “passenger” or a driver of CRC is a current debate.

## Oral bacteria in CRC pathogenesis

Bacterial species typically found in the oral cavity have been often found in the colon, though it is unknown whether these microbes originated in the oral cavity or whether particular strains are specialized to live in the colon.^61^ However, a study by Komiya et al.^62^ found that 40% of patients had identical strains of *F. nucleatum* in their CRC tumor tissue and saliva, postulating that *F. nucleatum* may originate in the oral cavity. A number of studies including a multicenter metagenome sequencing study across five countries have found that a number of oral cavity associated bacteria are over-represented in CRC, including *Fusobacterium, Porphyromonas, Parvimonas*, and *Prevotella*.^63^ Other studies have found that tumors with characteristics of the adenoma-carcinoma sequence, which typically develop from adenomas, were found to have increased abundance of *Capnocytophaga*,^48^ *Selenomonas*, and *Prevotella*.^49^ In contrast, the oral microbes *Fusobacterium nucleatum, Parvimonas micra, Peptostreptococcus stomatis* and *Porphyromonas gingivalis* were associated with Consensus Molecular Subtype 1 (CMS1) tumors,^49^ which are closely associated with the serrated pathway.^64^ This study also noted that these oral microbes are capable of forming biofilms, and that *P. gingivalis* co-aggregates with *Treponema denticola* and *Tannerella forsythia*, which are also enriched in CMS1 tumors.^49^ A number of previously mentioned studies also associate *Fusobacterium* with premalignant polyps with features of the serrated pathway. A study by Hale et al. found that MMR deficient CRC tumors, seen in the serrated pathway, were enriched with *Fusobacterium nucleatum* and *periodonticum*.^50^

It remains unknown whether alterations in the colonic mucosa attract oral flora associated microbes or if they become present upon the development of the CRC tumor environments. A 2018 study by Flemer et al. found that networks of typically oral bacteria were more abundant on polyps and CRC tumors than on the mucosa of healthy individuals. The microbial networks from polyps and CRC tumors were similar to those from the patient’s oral swabs as well as their healthy mucosa. They postulated that this may indicate that oral cavity-associated bacterial networks may exist prior to and contribute to the development of CRC.

Our group questions whether dietary habits could lead to changes in the gut epithelium, such as increased permeability or alterations in pH, that allow oral bacteria to survive in the intestines.^65^ Gliadin intake or a low fiber diet leading to reduced butyrate, crucial for the maintenance of cell adhesion and epithelial barrier defense, could in theory alter barrier function. Further, if colonocytes lack butyrate, they may switch to metabolizing other energy sources, such as carbohydrates. In line with this thinking, Flemer et al. found that when oral pathogens were present in the colonic mucosa, their presence was strongly negatively correlated with the abundance of butyrate-producer *Lachnospiraceae*. The abundance of *Lachnospiraceae* was weakly negatively correlated with a Western diet and was postulated to prevent colonic colonization by the typically oral bacteria that are associated with CRC. While there have not been any confirmed mechanisms by which these bacteria contribute to CRC carcinogenesis, some mechanisms have been proposed regarding communities of oral cavity associated bacteria found in biofilms. An example of an oral biofilm is dental plaque, which is colonized by *Streptococcus* and *Actinomyces*, creating an anaerobic environment conducive to *F. nucleatum* and other oral bacteria survival.^66^ Biofilms in the colon may also harbor oral cavity associated bacteria, including commensal bacteria and the pathogenic periodontal bacteria *F. nucleatum* and *P. gingivalis*.^67^ The new environment of the biofilm has been suggested to allow for opportunistic pathogens associated with the oral cavity, such as *Peptostreptococcus* and *Porphyromonas*, to survive and promote CRC tumorigenesis. Proposed mechanisms include bacterial secretion of enzymes that disrupt the protective mucus layer and destruction of defensin peptides and IgA antibodies, allowing bacteria to attach to and invade the mucosa and incite inflammation via IL-6, STAT3,^66^ and IL-8 pathways.^68^

## Conclusion

Research has established that the intestinal microbiome is different in patients with CRC. Most studies have utilized stool samples, and some have used mucosal samples. Of the studies that use mucosal samples, the comparison of the microbiome is often between an adenoma (without further specification of type) or tumor to normal tissue. However, there are very few studies that have investigated the microbiome of adenomas, SSPs, TSAs, and tumors in the adenoma-carcinoma sequence and serrated pathway specifically. This distinction can be made by specifying each sample’s genetic, epigenetic, or histopathologic type, or location in the colon as a proxy. Analysis of the select studies that have made these distinctions revealed that microbes typically considered normal oral flora can be found in the colons of patients with CRC and in biofilms, and that *Fusobacterium* is associated with right sided, more advanced, and serrated pathway lesions. One study suggested that the colons from people with CRC have more biofilms, particularly on proximal tumors.^53^ While these findings cannot yet change clinical practice, they indicate a possible future where microbial signatures may be used to screen for and characterize precursor polyps and be used to characterize which carcinogenic pathway they were derived from or might progress through. This level of detail could open the door for novel prevention, screening, and treatment interventions for CRC based on carcinogenic pathway characterization. Eventually, CRC interventions could expand to include microbial manipulation. For this to become a possibility, more clinical studies are required to investigate the microbiome of precursor polyps as well as tumors in each stage of carcinogenesis in the adenoma-carcinoma sequence and serrated pathways. This will require mucosal samples and characterization by histopathology, genetic and molecular profile, and location in the colon. Longitudinal studies should begin early on, prior to the occurrence of any colon pathology, and extend through CRC treatment and monitoring. If particular microbial populations or metabolites are associated with CRC carcinogenesis, further bench research will be necessary to investigate possible mechanisms of carcinogenesis and interventions. Specifically, experimental studies with model organisms and specific *in vitro* cell cultures that reflect the distinct pathways to CRC to investigate how oral cavity associated microbes and biofilms may further CRC progression. Finally, it is critical that microbiome researchers use consistent standards and make their data and analyses open access to support rigorous and reproducible science.^69,70–80^
